# Polydeoxyribonucleotide Enhances Liver Regeneration Following Partial Hepatectomy in Rats in Association with Growth Factor and A_2A_ Receptor Signaling

**DOI:** 10.3390/medicina62091819

**Published:** 2026-09-21

**Authors:** Il-Gyu Ko, Hyoung Il Choi, Su Bee Park, Hyun Phil Shin, Jung Won Jeon, SeungHwan Lee

**Affiliations:** 1 Research Support Center, School of Medicine, Keimyung University, Daegu 42601, Republic of Korea; rhdlfrb@naver.com; 2 Department of Internal Medicine, Kyung Hee University Hospital at Gangdong, College of Medicine, Kyung Hee University, Seoul 05278, Republic of Korea; fullhouse11245@naver.com (H.I.C.); endobee90@gmail.com (S.B.P.); megadoctor@khnmc.or.kr (H.P.S.); drglory@naver.com (J.W.J.); 3 Department of Surgery, Kyung Hee University Hospital at Gangdong, College of Medicine, Kyung Hee University, Seoul 05278, Republic of Korea

**Keywords:** polydeoxyribonucleotide, partial hepatectomy, liver regeneration, hepatocyte proliferation, adenosine A_2A_ receptor

## Abstract

*Background and Objectives*: Liver regeneration following partial hepatectomy (PHx) is essential for restoring hepatic function. Polydeoxyribonucleotide (PDRN), an adenosine A_2A_ receptor (A_2A_R) agonist, has demonstrated regenerative and proangiogenic properties in various tissues. However, its therapeutic potential for promoting liver regeneration after PHx has not been fully elucidated. The primary objective was to evaluate the effect of PDRN on liver regeneration after PHx, and the secondary objective was to examine associated changes in proliferation-related proteins and A_2A_R/cAMP, HGF/c-Met, and VEGF/VEGFR2 signaling. *Materials and Methods*: A rat model of 70% PHx was established, and PDRN (8 mg/kg) was administered intraperitoneally once daily for 5 consecutive days with or without the A_2A_R antagonist 3,7-dimethyl-1-propargylxanthine (DMPX; 8 mg/kg). Liver regeneration was evaluated using biochemical, histological, and molecular analyses. *Results*: PDRN enhanced liver regeneration following PHx, as demonstrated by reduced serum liver enzyme levels, enhanced recovery of liver mass, and improved histological architecture. PDRN also increased the expression of the proliferation-associated proteins proliferating cell nuclear antigen (PCNA) and cyclin D1. These effects were accompanied by increased A_2A_R expression and cyclic adenosine monophosphate (cAMP) levels, elevated hepatocyte growth factor (HGF) and vascular endothelial growth factor (VEGF) levels, and enhanced phosphorylation of c-Met and VEGFR2. Co-administration of DMPX attenuated the regenerative and molecular effects of PDRN, suggesting a potential involvement of A_2A_R-related signaling. *Conclusions*: Collectively, these findings suggest that PDRN may facilitate liver regeneration following PHx in association with increased expression of proliferation-associated proteins and changes in HGF/c-Met and VEGF/VEGFR2 signaling, supporting its potential as a therapeutic strategy following hepatic resection.

## 1. Introduction

The liver possesses a remarkable regenerative capacity, enabling it to restore its mass and function following injury or surgical resection. Partial hepatectomy (PHx) has long served as a representative experimental model for investigating the mechanisms of liver regeneration because it induces a highly coordinated regenerative response involving hepatocyte proliferation, extracellular matrix remodeling, and restoration of hepatic function [1,2]. Although liver regeneration is generally efficient under physiological conditions, regenerative responses may be impaired following extensive hepatic resection or in patients with chronic liver disease, thereby increasing the risk of postoperative liver failure and poor clinical outcomes [3,4]. Therefore, identifying pharmacological agents that enhance liver regeneration remains an important therapeutic objective.

Liver regeneration is orchestrated by a complex network of cytokines, growth factors, and intracellular signaling pathways. Following PHx, hepatocyte proliferation requires coordinated cell-cycle progression, which can be assessed by proliferation-associated proteins such as proliferating cell nuclear antigen (PCNA) and cyclin D1 [5,6]. Among the growth factors involved in liver regeneration, hepatocyte growth factor (HGF) is recognized as one of the principal mitogens responsible for initiating hepatocyte proliferation through activation of the c-Met receptor, whereas vascular endothelial growth factor (VEGF) contributes to angiogenesis and vascular remodeling, thereby supporting regeneration of the hepatic microenvironment [7,8,9]. In addition to these classical growth factors, extracellular adenosine has emerged as an important endogenous mediator of tissue protection and regeneration. Activation of the adenosine A_2A_ receptor (A_2A_R) stimulates intracellular cyclic adenosine monophosphate (cAMP) production and regulates signaling pathways involved in tissue repair and regenerative responses [10,11,12,13]. However, the contribution of A_2A_R-mediated signaling to liver regeneration following PHx remains incompletely understood.

Polydeoxyribonucleotide (PDRN), a mixture of deoxyribonucleotide polymers extracted from trout sperm, has attracted considerable attention because of its tissue-protective, anti-inflammatory, and regenerative properties. PDRN exerts its biological effects primarily through activation of the A_2A_R, resulting in increased cAMP production, suppression of inflammatory responses, and stimulation of VEGF-dependent tissue repair [14,15]. Experimental studies have also demonstrated beneficial effects of PDRN in various experimental disease models [16,17].

Despite these findings, the therapeutic potential and underlying mechanisms of PDRN in liver regeneration following PHx have not yet been fully elucidated. Therefore, the present study investigated whether PDRN enhances liver regeneration following PHx in rats. We also examined its effects on proliferation-associated proteins and the A_2A_R/cAMP, HGF/c-Met, and VEGF/VEGFR2 signaling pathways.

## 2. Materials and Methods

### 2.1. Experimental Animals and Grouping

Male Sprague-Dawley rats (10 weeks of age, weighing 250 ± 10 g) were obtained from Orient Bio Inc. (Seongnam, Republic of Korea). The animals were housed under controlled environmental conditions (23 ± 2 °C, 55 ± 5% humidity, and a 12-h light/12-h dark cycle) with free access to standard laboratory chow and water throughout the experimental period. All animals were allowed to acclimate for 1 week before the experiments began. All experimental procedures were approved by the Institutional Animal Care and Use Committee of Kyung Hee University (KHUASP[SE]-21-495) and were conducted in accordance with established guidelines for the care and use of laboratory animals. For the PHx groups, animals with inadequate postoperative recovery were excluded before treatment group allocation. Eligible animals were subsequently randomly divided among the three PHx-related experimental groups (*n* = 10 per group): PHx-induced group (PHx), PHx-induced and PDRN-treated group (PHx-PDRN), and PHx-induced and PDRN+DMPX-treated group (PHx-PDRN+DMPX). The sham-operated group consisted of 10 animals. Outcome assessments were performed by investigators blinded to the treatment groups, except for the investigator responsible for drug administration.

### 2.2. Partial Hepatectomy

**Figure 1:**
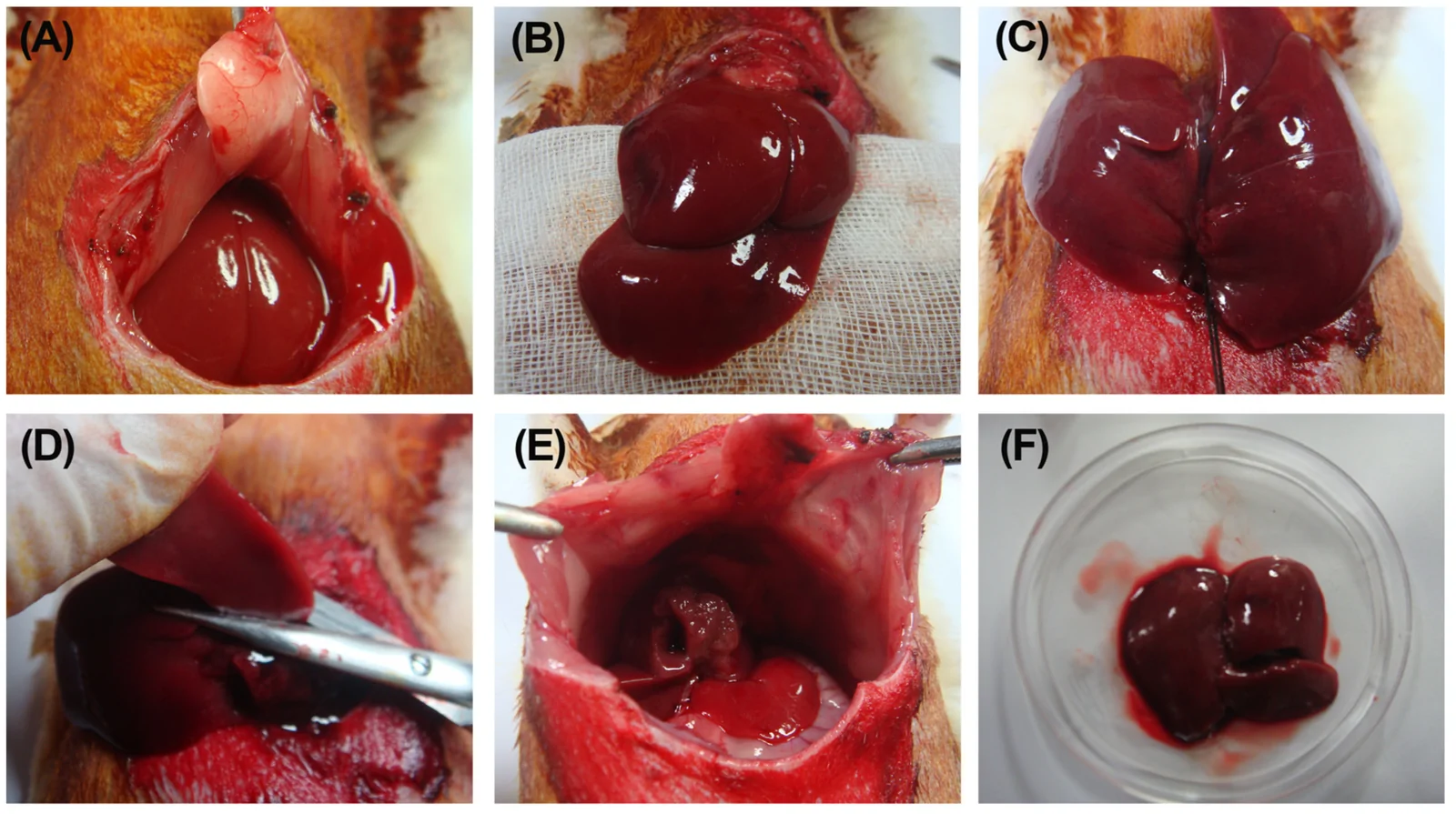
Surgical procedure for 70% partial hepatectomy in rats. (**A**) Initial exposure of the liver following midline laparotomy. (**B**) Exteriorization of the liver. (**C**) Ligation of the liver lobes designated for resection. (**D**) Resection of the ligated liver lobes. (**E**) Remnant liver immediately following partial hepatectomy. (**F**) Resected liver lobes.

A 70% PHx was performed using a modified version of previously described methods [18]. After anesthesia was induced with 2% isoflurane (30% oxygen and 70% nitrogen), the abdomen was shaved and disinfected with povidone-iodine. A midline laparotomy was performed under sterile conditions to expose the liver. The median and left lateral lobes, corresponding to approximately 70% of the total liver mass, were carefully mobilized and ligated en masse at their pedicles using 1-0 silk sutures. The liver lobes were then resected distal to the ligation, and the resected lobes were weighed immediately after removal. Meticulous hemostasis was confirmed before abdominal closure. The abdominal muscle and skin were closed separately using absorbable sutures. Body temperature was maintained at 36 ± 0.5 °C throughout surgery using a Homeothermic Blanket Control Unit (Harvard Apparatus, Holliston, MA, USA). After surgery, the animals were monitored for 2 h while recovering from anesthesia to prevent hypothermia. Sham-operated animals underwent the same surgical procedures without liver resection. The major steps of the 70% PHx procedure are shown in Figure 1.

### 2.3. Drug Administration

**Figure 2:**
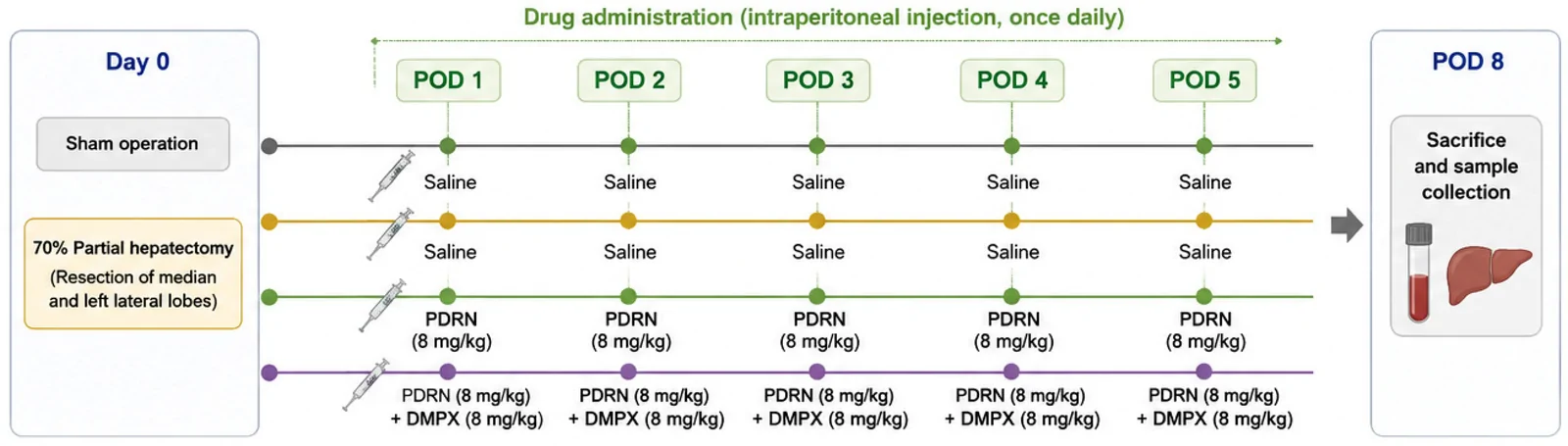
Experimental design and treatment schedule. POD, postoperative day; PDRN, polydeoxyribonucleotide; DMPX, 3,7-dimethyl-1-propargylxanthine.

PDRN (Rejuvenex^®^, PharmaResearch Products Co., Seongnam, Republic of Korea) was administered intraperitoneally at a dose of 8 mg/kg once daily for 5 consecutive days, beginning on postoperative day 1. To investigate the involvement of A_2A_R signaling, rats in the PHx-PDRN+DMPX group received DMPX (8 mg/kg, i.p.; Sigma-Aldrich, St. Louis, MO, USA) concurrently with PDRN. Rats in the remaining groups received an equivalent volume of normal saline. The experimental timeline is shown in Figure 2.

### 2.4. Sample Collection and Liver Mass Assessment

On postoperative day 8, the rats were anesthetized with isoflurane, and blood samples were collected from the abdominal aorta. Following exsanguination, the animals were euthanized, and the remnant liver was immediately excised and weighed. Body weight was measured immediately before euthanasia. Liver mass recovery was assessed by measuring the remnant liver weight and calculating the liver weight-to-body weight ratio (LW/BW). The remnant liver was then divided for histological and biochemical analyses. Portions of the liver were fixed in 10% neutral-buffered formalin for histological examination, whereas the remaining tissues were snap-frozen in liquid nitrogen and stored at −70 °C until further analysis.

### 2.5. Serum Biochemical Analysis

Blood samples were centrifuged at 3000× *g* for 15 min to obtain serum. Serum samples were stored at −70 °C until analysis. Serum levels of aspartate aminotransferase (AST), alanine aminotransferase (ALT), lactate dehydrogenase (LDH), alkaline phosphatase (ALP), albumin, total bilirubin, and direct bilirubin were measured using an automated biochemical analyzer (AU400; Olympus, Tokyo, Japan).

### 2.6. Histopathological Examination

Liver tissues were fixed in 10% neutral-buffered formalin, embedded in paraffin, sectioned at 5 μm, and stained with hematoxylin and eosin (H&E). Histological changes were examined under a light microscope (Olympus, Tokyo, Japan). A semiquantitative histological scoring system based on previous liver regeneration studies was used to evaluate hepatocyte degeneration, sinusoidal dilatation, inflammatory cell infiltration, and restoration of hepatic architecture [19,20]. Each parameter was graded on a scale of 0–4 (0, absent; 1, minimal; 2, mild; 3, moderate; and 4, severe), and the total histological score was calculated. Histological assessment was performed independently by three investigators blinded to the experimental groups, and the mean score was used for statistical analysis.

### 2.7. Enzyme-Linked Immunosorbent Assay (ELISA)

Liver tissues were homogenized in ice-cold lysis buffer and centrifuged at 12,000× *g* for 20 min at 4 °C. The supernatants were collected and stored at −70 °C until analysis. The tissue concentrations of A_2A_R (LS-F34417-1; LSBio, Seattle, WA, USA), cAMP (ab234585; Abcam, Cambridge, UK), VEGF (ab100786; Abcam), and HGF (LS-F33066-1; LSBio) were determined using commercially available ELISA kits according to the manufacturers’ instructions. The assay sensitivity was 0.1 nM for cAMP and <2 pg/mL for VEGF, with detection ranges of 0.82–200 pg/mL for VEGF and 62.5–4000 pg/mL for HGF. Absorbance was measured using a microplate reader, and concentrations were calculated from the standard curves.

### 2.8. Western Blot Analysis

Western blot analysis was performed as previously described [21,22]. Liver tissues were homogenized in lysis buffer containing 50 mM Tris-HCl (pH 8.0), 150 mM NaCl, 10% glycerol, 1% Triton X-100, 1.5 mM MgCl_2_·6H_2_O, 1 mM EGTA, 1 mM phenylmethylsulfonyl fluoride (PMSF), 1 mM Na_2_VO_4_, and 100 mM NaF and then centrifuged at 14,000 rpm for 30 min. Protein concentrations were determined using a protein assay kit (Bio-Rad Laboratories, Hercules, CA, USA). Equal amounts of protein (30 μg) were separated by SDS-polyacrylamide gel electrophoresis and transferred onto nitrocellulose membranes. The membranes were incubated overnight at 4 °C with primary antibodies against PCNA (#2586, 1:1000; Cell Signaling Technology, Danvers, MA, USA), cyclin D1 (#55506, 1:1000; Cell Signaling Technology), A_2A_R (ab3461, 1:1000; Abcam), VEGFR2 (#9698, 1:1000; Cell Signaling Technology), p-VEGFR2 (#2478, 1:1000; Cell Signaling Technology), c-Met (#3127, 1:1000; Cell Signaling Technology), p-c-Met (#3077, 1:1000; Cell Signaling Technology), and β-actin (#4970, 1:1000; Cell Signaling Technology). After washing, the membranes were incubated with horseradish peroxidase (HRP)-conjugated secondary antibodies (PI-1000-1 and PI-2000-1, 1:3000; Vector Laboratories). Protein bands were visualized using an enhanced chemiluminescence (ECL) detection kit (Santa Cruz Biotechnology, Dallas, TX, USA) and quantified by densitometric analysis using Molecular Analyst™ software (Bio-Rad Laboratories). Western blot analyses were performed using liver samples from five independent animals per group (n = 5), with repeated measurements not considered additional biological replicates.

### 2.9. Statistical Analysis

Histological evaluation was performed independently by three investigators blinded to the experimental groups, and the average score was used for statistical analysis. Data are presented as the mean ± SEM. Statistical analyses were performed using SPSS version 23.0 (IBM Corp., Armonk, NY, USA). The liver-to-body weight ratio was considered the primary outcome for liver mass recovery. Normality and homogeneity of variance were assessed before parametric analysis. Continuous variables were analyzed using one-way ANOVA followed by Duncan’s multiple range test, whereas histological scores were analyzed using the Kruskal-Wallis test followed by Holm-adjusted Mann-Whitney U tests for pairwise comparisons. A *p* value < 0.05 was considered statistically significant. Detailed statistical results are provided in Appendix Table A1.

## 3. Results

### 3.1. PDRN Promoted Liver Regeneration Following PHx

**Figure 3:**
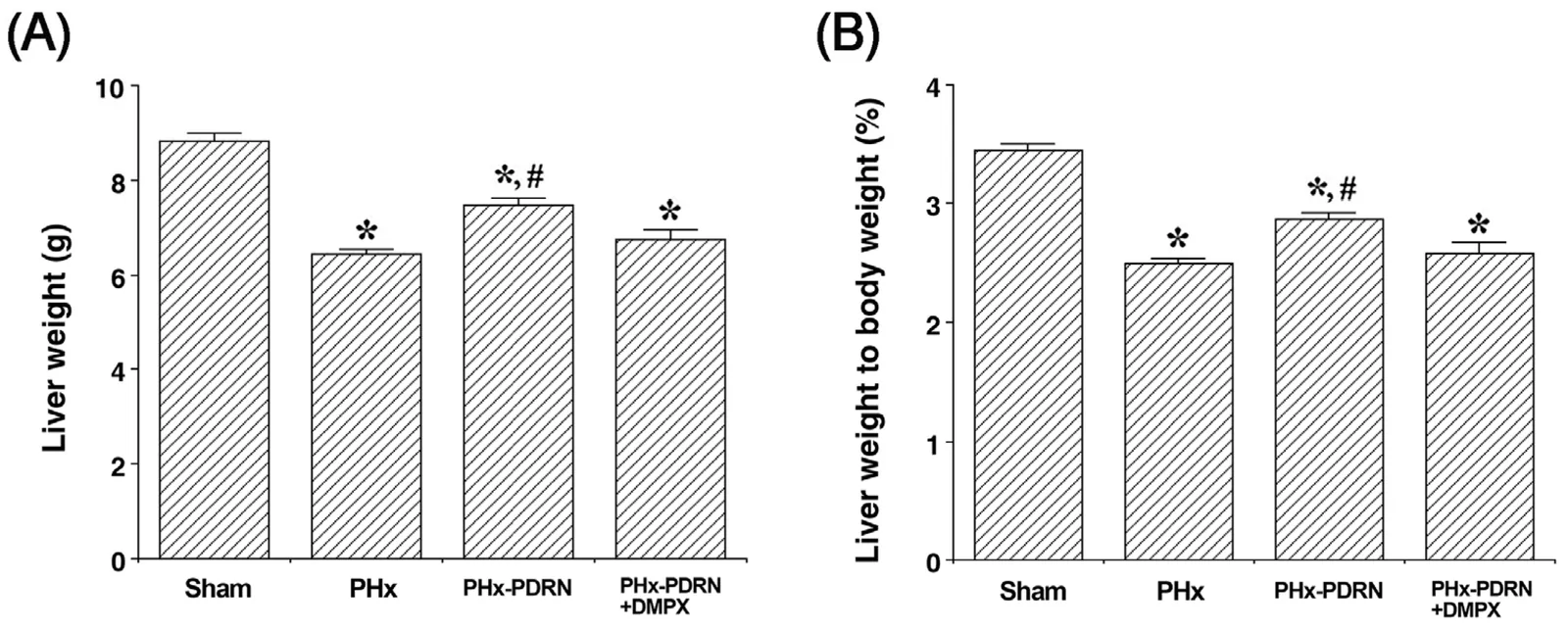
Liver weight and liver-to-body weight ratio following partial hepatectomy. (**A**) Liver weight, (**B**) Liver-to-body weight ratio. (Sham) Sham-operated group, (PHx) PHx-induced group, (PHx-PDRN) PHx-induced and PDRN-treated group, (PHx-PDRN+DMPX) PHx-induced and PDRN+DMPX-treated group. Data are presented as mean ± SEM. * *p* < 0.05 versus the sham-operated group; # *p* < 0.05 versus the PHx-induced group.

Recovery of liver mass was evaluated by measuring the remnant liver weight and LW/BW ratio on postoperative day 8. The mean resected liver weight at the time of PHx was 6.71 ± 0.07 g among the 40 animals undergoing PHx (Appendix Table A2). Compared with the sham-operated group, the PHx group had significantly lower liver weight and a lower LW/BW ratio. PDRN treatment significantly increased both parameters compared with those in the PHx group, indicating enhanced recovery of liver mass following PHx. In contrast, co-administration of DMPX attenuated these effects, resulting in significantly lower liver weight and a lower LW/BW ratio than those in the PHx-PDRN group (Figure 3).

### 3.2. PDRN Improved Serum Biochemical Parameters Following PHx

**Figure 4:**
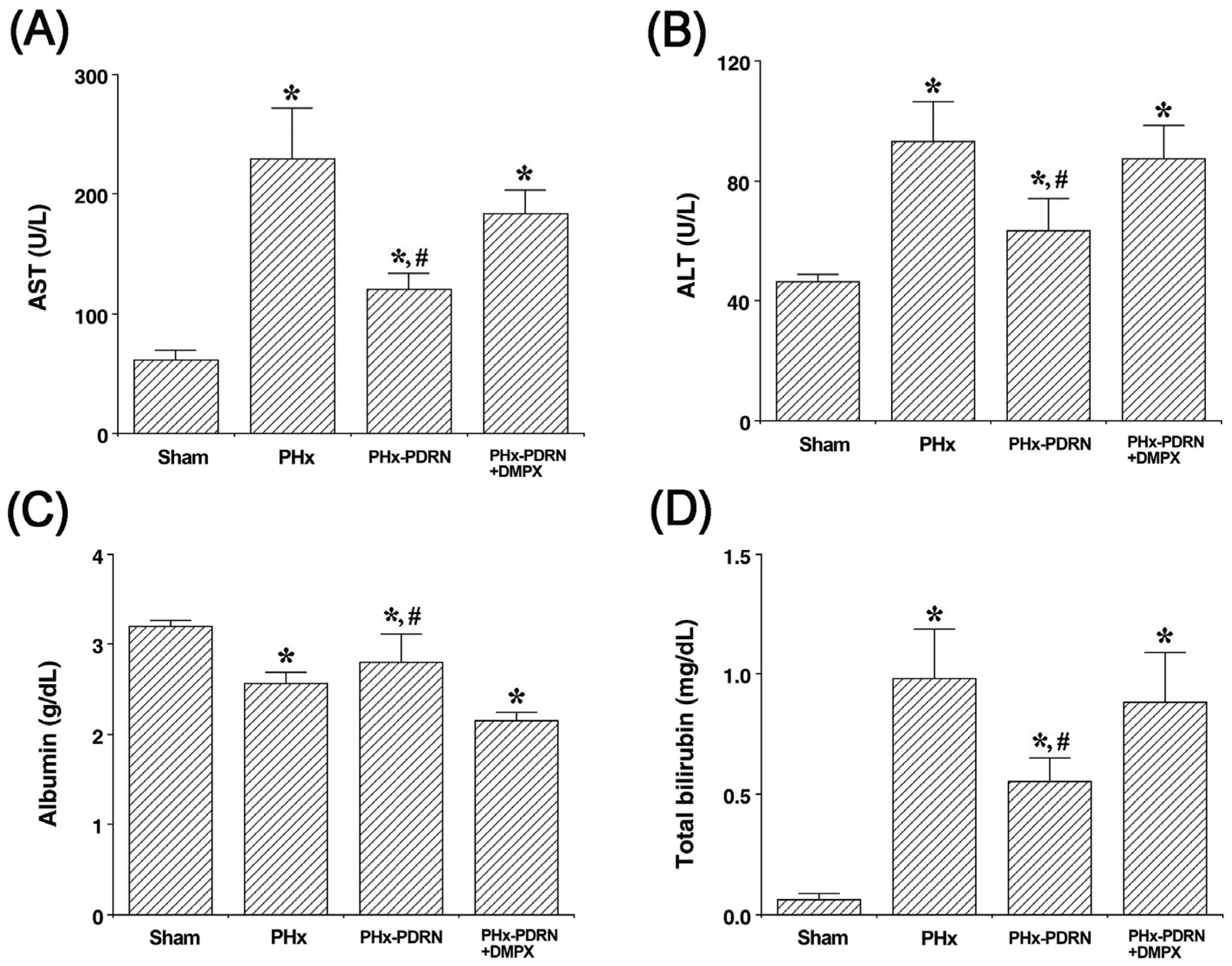
Serum biochemical parameters following partial hepatectomy. (**A**) Aspartate aminotransferase; AST, (**B**) Alanine aminotransferase; ALT, (**C**) Albumin, (**D**) Total bilirubin. (Sham) Sham-operated group, (PHx) PHx-induced group, (PHx-PDRN) PHx-induced and PDRN-treated group, (PHx-PDRN+DMPX) PHx-induced and PDRN+DMPX-treated group. Data are presented as mean ± SEM. * *p* < 0.05 versus the sham-operated group; # *p* < 0.05 versus the PHx-induced group.

**Figure A1:**
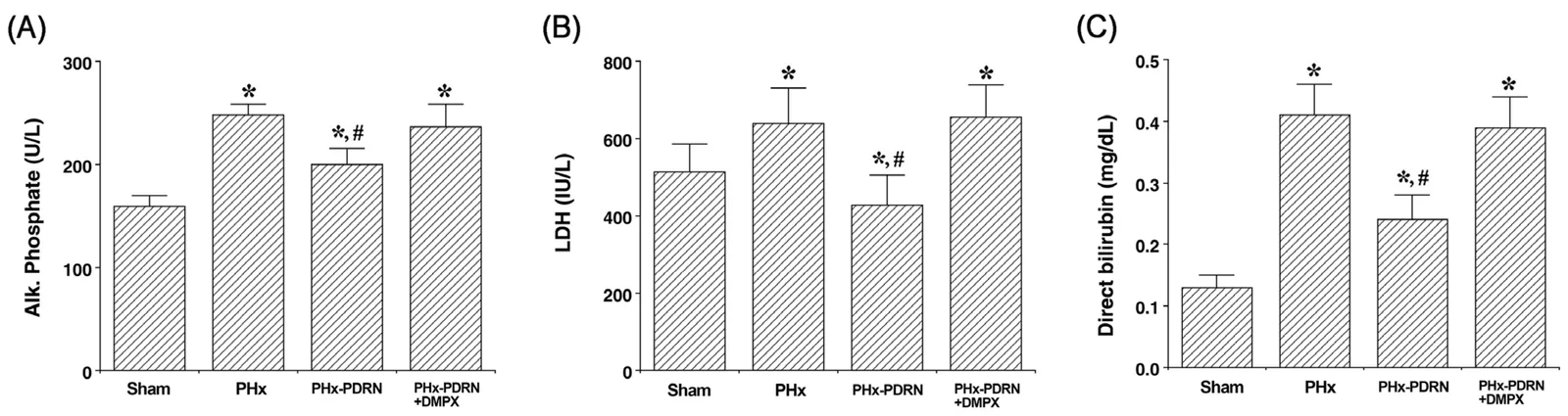
Effects of PDRN on additional serum biochemical parameters following partial hepatectomy. (**A**) Serum alkaline phosphatase (ALP), (**B**) lactate dehydrogenase (LDH), and (**C**) direct bilirubin levels. Data are presented as mean ± standard error of the mean. * *p* < 0.05 versus the sham-operated group; # *p* < 0.05 versus the PHx-induced group.

Serum biochemical parameters were evaluated to assess liver function following PHx. Compared with the sham-operated group, the PHx group exhibited significantly increased serum AST and ALT levels, accompanied by decreased albumin and increased total bilirubin levels. PDRN treatment significantly reduced serum AST and ALT levels while restoring albumin and total bilirubin toward the levels observed in the sham-operated group. These effects were attenuated by co-administration of DMPX (Figure 4). In addition, similar trends were observed for serum ALP, LDH, and direct bilirubin levels (Figure A1).

### 3.3. PDRN Ameliorated Histological Alterations Following PHx

**Figure 5:**
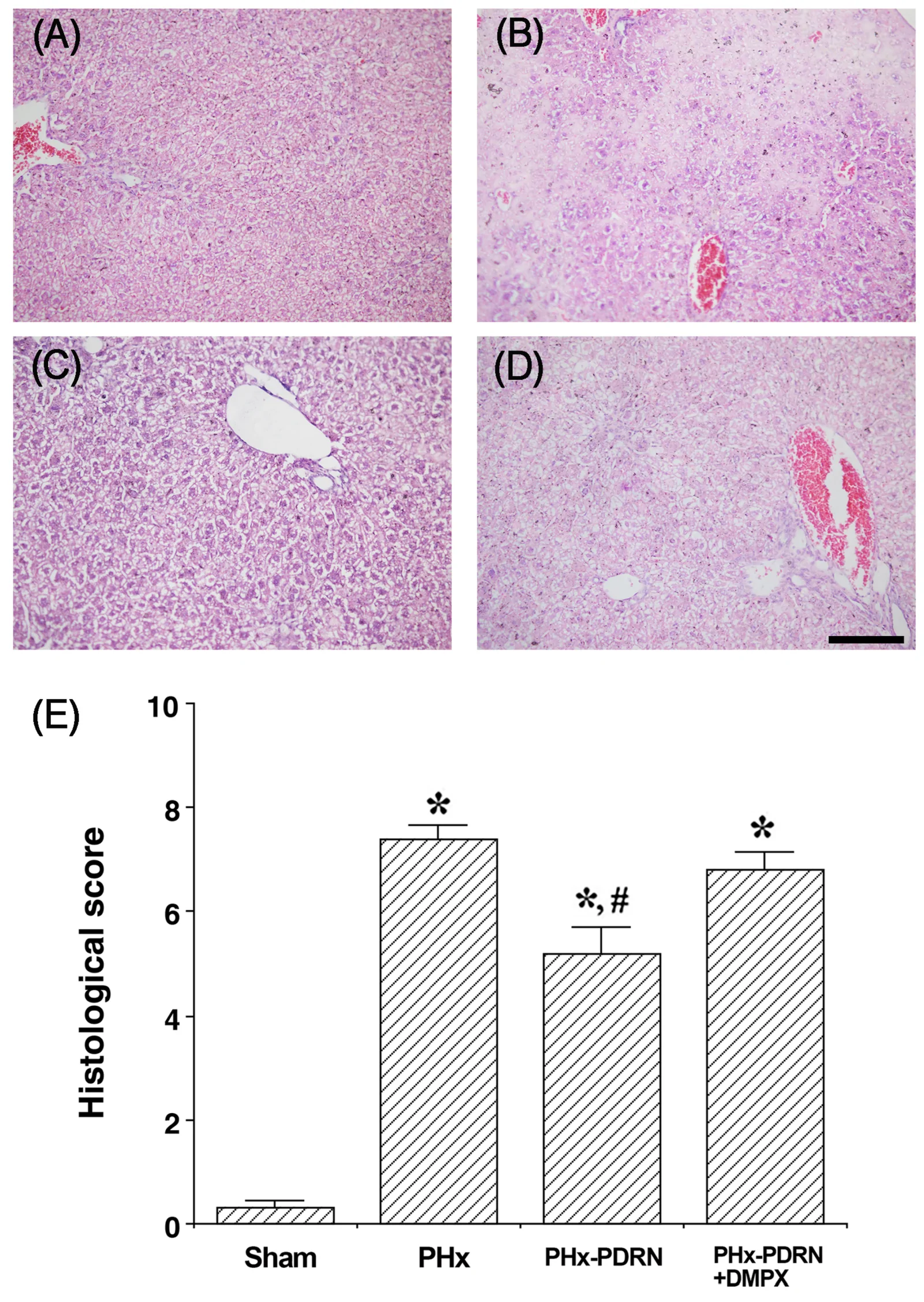
Histological changes in the liver following partial hepatectomy. (**A**–**D**) Representative hematoxylin and eosin (H&E)-stained liver sections from the experimental groups: (**A**) Sham-operated group, (**B**) PHx-induced group, (**C**) PHx-induced and PDRN-treated group, (**D**) PHx-induced and PDRN+DMPX-treated group. The scale bar represents 150 μm. (**E**) Histological scores in each group. Data are presented as mean ± SEM. Histological scores were analyzed using the Kruskal-Wallis test followed by Holm-adjusted Mann-Whitney U tests for pairwise comparisons. * *p* < 0.05 versus the sham-operated group; # *p* < 0.05 versus the PHx-induced group.

Histological changes in the remnant liver were evaluated by H&E staining on postoperative day 8. The sham-operated group exhibited preserved hepatic architecture with relatively uniform hepatocyte organization. In contrast, the PHx group showed marked morphological alterations, including irregular hepatocyte organization and disruption of the sinusoidal architecture. These changes were partially ameliorated by PDRN treatment, whereas co-administration of DMPX diminished the histological improvement induced by PDRN (Figure 5). The overall difference among the groups was significant (Kruskal-Wallis test, *p* < 0.001), and the relevant pairwise comparisons remained significant after adjustment for multiple comparisons.

Consistent with these observations, the histological score was significantly increased in the PHx group compared with the sham-operated group. PDRN significantly reduced the histological score, whereas co-administration of DMPX attenuated this reduction (Figure 5).

### 3.4. PDRN Increased the Expression of Proliferation-Associated Proteins Following PHx

**Figure 6:**
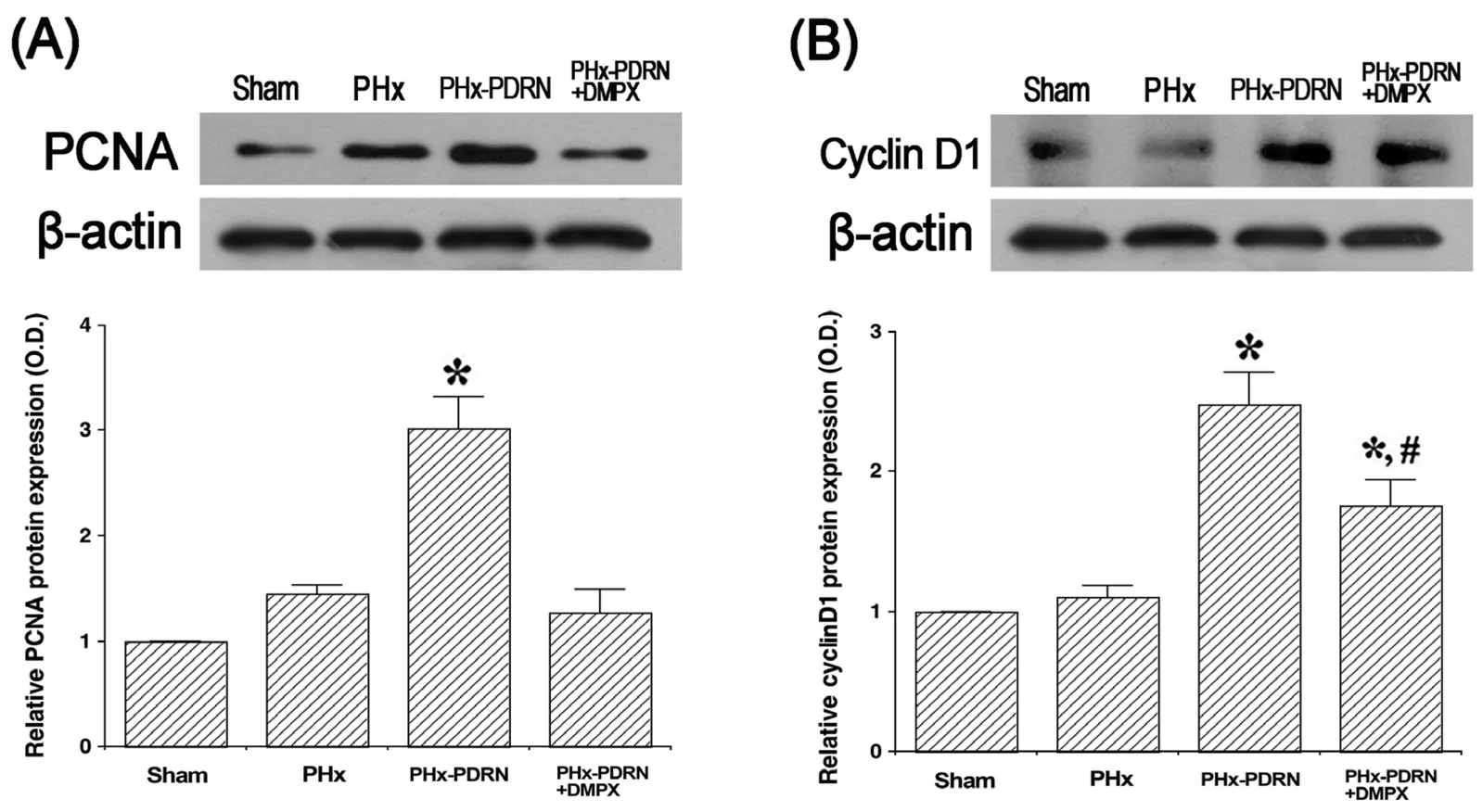
Expression of proliferation-associated proteins following partial hepatectomy. (**A**) Representative western blot images and quantitative analysis of PCNA expression in liver tissue. (**B**) Representative western blot images and quantitative analysis of cyclin D1 expression in liver tissue. β-actin was used as an internal control (46 kDa). (Sham) Sham-operated group, (PHx) PHx-induced group, (PHx-PDRN) PHx-induced and PDRN-treated group, (PHx-PDRN+DMPX) PHx-induced and PDRN+DMPX-treated group. Data are presented as mean ± SEM. * *p* < 0.05 versus the sham-operated group; # *p* < 0.05 versus the PHx-induced group.

Western blot analysis was performed to assess the expression of the proliferation-associated proteins PCNA and cyclin D1. PCNA and cyclin D1 expression showed only modest changes in the PHx group compared with the sham-operated group, whereas both proteins were markedly increased following PDRN treatment. PCNA expression was substantially reduced by co-administration of DMPX, approaching the level observed in the PHx group. Cyclin D1 expression was also significantly reduced by DMPX compared with the PDRN-treated group, although it remained elevated relative to the sham-operated group (Figure 6). These findings indicate that PDRN increased the expression of proliferation-associated proteins following PHx and that these increases were attenuated by co-administration of DMPX.

### 3.5. PDRN Increased A_2A_R Expression and cAMP Levels Following PHx

**Figure 7:**
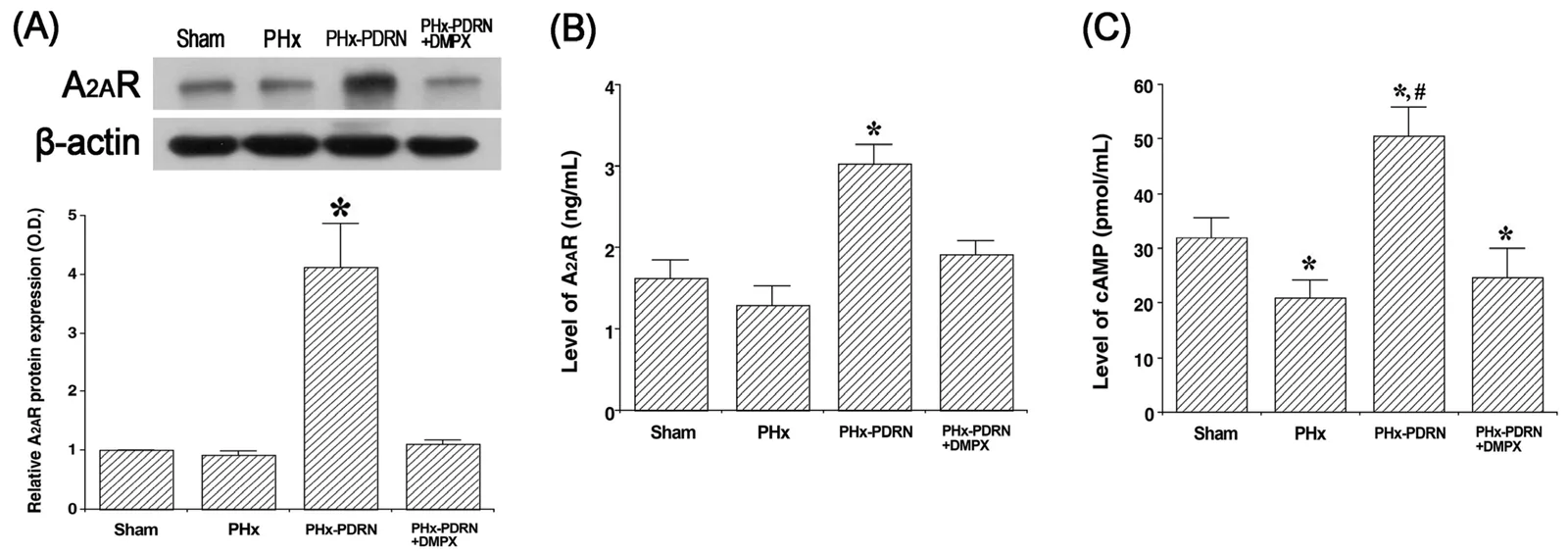
A_2A_R/cAMP signaling following partial hepatectomy. (**A**) Representative Western blot images and quantitative analysis of A_2A_R expression in liver tissue. β-actin was used as an internal control (46 kDa). (**B**) A_2A_R levels were determined by ELISA. (**C**) cAMP levels were determined by ELISA. (Sham) Sham-operated group, (PHx) PHx-induced group, (PHx-PDRN) PHx-induced and PDRN-treated group, (PHx-PDRN+DMPX) PHx-induced and PDRN+DMPX-treated group. Data are presented as mean ± SEM. * *p* < 0.05 versus the sham-operated group; # *p* < 0.05 versus the PHx-induced group.

To investigate whether the regenerative effects of PDRN were associated with A_2A_R signaling, hepatic A_2A_R expression and cAMP levels were evaluated. Compared with those in the sham-operated group, cAMP levels were significantly decreased in the PHx group, whereas A_2A_R expression showed no significant changes. PDRN treatment significantly increased A_2A_R expression and restored hepatic cAMP levels compared with the PHx group. In contrast, co-administration of DMPX attenuated these effects, resulting in significantly lower A_2A_R expression and cAMP levels than those in the PDRN-treated group (Figure 7A–C). These findings indicate that the regenerative effects of PDRN were accompanied by increased A_2A_R expression and cAMP levels following PHx.

### 3.6. PDRN Increased VEGF/HGF Levels and Receptor Phosphorylation Following PHx

**Figure 8:**
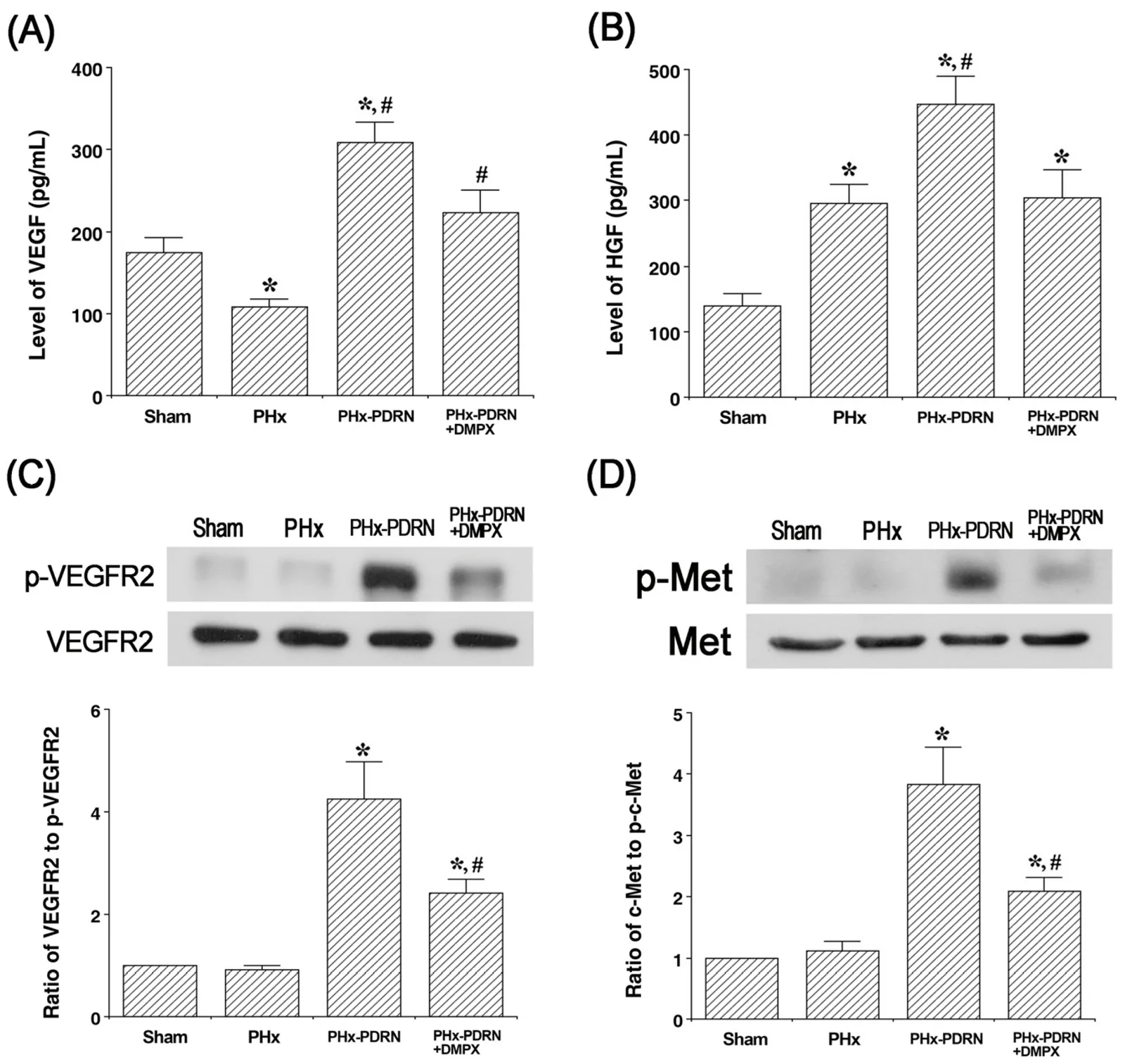
VEGF/VEGFR2 and HGF/c-Met signaling following partial hepatectomy. (**A**) VEGF levels were determined by ELISA. (**B**) HGF levels were determined by ELISA. (**C**) Representative Western blot images and quantitative analysis of VEGFR2 phosphorylation in liver tissue. (**D**) Representative Western blot images and quantitative analysis of c-Met phosphorylation in liver tissue. (Sham) Sham-operated group, (PHx) PHx-induced group, (PHx-PDRN) PHx-induced and PDRN-treated group, (PHx-PDRN+DMPX) PHx-induced and PDRN+DMPX-treated group. Data are presented as mean ± SEM. * *p* < 0.05 versus the sham-operated group; # *p* < 0.05 versus the PHx-induced group.

To further investigate molecular changes associated with the regenerative effects of PDRN, hepatic VEGF and HGF levels and the phosphorylation of their downstream receptors were evaluated. Compared with the PHx group, the PDRN-treated group displayed significantly increased hepatic VEGF and HGF levels. These effects were attenuated by co-administration of DMPX (Figure 8A,B).

Western blot analysis demonstrated that PDRN significantly enhanced the phosphorylation of VEGFR2 and c-Met compared with that in the PHx group, whereas total VEGFR2 and c-Met protein expression remained unchanged among the experimental groups. The PDRN-induced increase in the phosphorylation of both receptors was markedly attenuated following DMPX administration (Figure 8C,D). These findings indicate that the regenerative effects of PDRN were accompanied by increased VEGF/HGF-associated signaling following PHx.

## 4. Discussion

PHx is a standard treatment for many hepatic diseases; however, the inevitable loss of functional liver mass immediately after surgery necessitates rapid and effective liver regeneration to restore hepatic homeostasis [23,24]. In the present study, PDRN promoted liver recovery following PHx, as demonstrated by increased liver weight and liver-to-body weight ratio, improved serum biochemical parameters, and ameliorated histopathological alterations. Notably, the recovery of serum biochemical markers, particularly albumin and transaminase levels, indicates that PDRN attenuated hepatocellular injury and supported the restoration of hepatic synthetic and metabolic functions. These findings suggest that PDRN facilitates structural and functional recovery beyond the early proliferative phase of liver regeneration following PHx. Effective liver regeneration following PHx depends on the rapid entry of quiescent hepatocytes into the cell cycle, enabling restoration of the lost liver mass while maintaining hepatic function [25,26].

PCNA is closely associated with DNA replication and is widely used as a marker of proliferative activity, whereas cyclin D1 plays an important role in cell-cycle progression during liver regeneration [5,27]. In the present study, PDRN increased the expression of both PCNA and cyclin D1 following PHx, whereas these increases were attenuated by DMPX. These findings suggest that PDRN enhances proliferation-associated responses during liver regeneration. The attenuation of these responses by DMPX suggests a possible involvement of A_2A_R-related signaling, although this interpretation is limited by the absence of a PHx+DMPX group.

Liver regeneration is orchestrated by multiple molecular pathways that regulate hepatocyte proliferation and tissue repair [28]. Hepatocyte proliferation following PHx is tightly regulated by a complex network of growth factor-mediated signaling pathways [24]. Toll-like receptor (TLR) signaling also contributes to the regulation of liver regeneration following PHx. In particular, TLR3 has been shown to promote hepatocyte proliferation by facilitating the release of HGF from the hepatic extracellular matrix [29].

Among the growth factors involved in liver regeneration, HGF is widely recognized as the principal mitogen responsible for initiating hepatocyte proliferation through activation of its receptor, c-Met [30,31,32]. Activation of the HGF/c-Met signaling pathway promotes hepatocyte cell-cycle progression and contributes to the restoration of liver mass after PHx [31,33]. In the present study, HGF levels were increased following PHx, consistent with an endogenous regenerative response, whereas c-Met phosphorylation showed no significant change compared with the sham group. Notably, PDRN treatment further increased hepatic HGF levels and c-Met phosphorylation, indicating an association between PDRN treatment and HGF/c-Met signaling during liver regeneration following PHx. In addition to its effects on proliferation-associated responses, the regenerative activity of PDRN is closely associated with its well-established proangiogenic properties [34]. Previous studies have reported that PDRN increases VEGF expression and promotes angiogenesis in various experimental models, with these effects attenuated by DMPX [15,17]. While previous studies have primarily focused on VEGF expression, the biological effects of VEGF are largely mediated through VEGFR2, the principal receptor responsible for angiogenic signaling [35,36]. In the regenerating liver, VEGFR2 signaling in liver sinusoidal endothelial cells is essential for normal hepatocyte proliferation, and endothelial VEGFR2 deficiency markedly impairs liver regeneration following PHx [9]. Consistent with these observations, VEGF levels were reduced following PHx, whereas VEGFR2 phosphorylation showed no significant change compared with the sham group. In contrast, PDRN treatment markedly increased VEGF levels and VEGFR2 phosphorylation. These findings demonstrate that PDRN treatment was accompanied by increased VEGF levels and VEGFR2 phosphorylation, suggesting an association between PDRN treatment and VEGF/VEGFR2 signaling during liver regeneration following PHx. Furthermore, PDRN treatment significantly increased A_2A_R expression and cAMP levels in regenerating liver tissue. Although the precise relationships among A_2A_R/cAMP, HGF/c-Met, and VEGF/VEGFR2 signaling remain to be clarified, the concurrent changes observed in these pathways suggest that they may be associated with the regenerative effects of PDRN following PHx.

Together, these findings indicate that improved liver recovery following PDRN treatment was accompanied by increased proliferation-associated responses and changes in regenerative signaling pathways. Although the present findings provide mechanistic insight into the regenerative effects of PDRN, causal relationships among these signaling pathways were not established. The absence of a PHx+DMPX group limits definitive interpretation of A_2A_R involvement and represents a limitation of the present study. In addition, PCNA and cyclin D1 were evaluated as proliferation-associated proteins without a direct cellular assessment of hepatocyte proliferation, and angiogenesis was not directly assessed. Moreover, because only a single postoperative time point was examined, the present study could not determine whether PDRN altered the timing or magnitude of the regenerative response. Further studies are warranted to clarify the molecular interactions through which PDRN enhances liver regeneration following PHx. Beyond mechanistic studies, advances in artificial intelligence and image processing may also facilitate the evaluation of liver diseases through improved image-based diagnosis, treatment planning, and prognostic assessment.

## 5. Conclusions

In conclusion, PDRN enhanced liver regeneration following PHx, as evidenced by improved structural recovery and increased proliferation-associated responses. Improved liver recovery following PDRN treatment was accompanied by changes in HGF/c-Met and VEGF/VEGFR2 signaling, along with increased A_2A_R expression and cAMP levels. Although causal relationships among these pathways remain to be established, the observed molecular changes suggest their potential association with the regenerative effects of PDRN following PHx and support further investigation of PDRN as a potential therapeutic strategy following hepatic resection.

## Data Availability

The data are presented in the manuscript. Additional information obtained during the experiments is available upon request.

## Abbreviations

The following abbreviations are used in this manuscript:

A_2A_R	Adenosine A_2A_ receptor
ALT	Alanine aminotransferase
ALP	Alkaline phosphatase
AST	Aspartate aminotransferase
cAMP	cyclic adenosine monophosphate
DMPX	3,7-dimethyl-1-propargylxanthine
ELISA	Enzyme-linked immunosorbent assay
HGF	Hepatocyte growth factor
LDH	Lactate dehydrogenase
LW/BW	Liver weight-to-body weight ratio
PCNA	Proliferating cell nuclear antigen
PDRN	Polydeoxyribonucleotide
PHx	Partial hepatectomy
VEGF	Vascular endothelial growth factor
VEGFR2	Vascular endothelial growth factor receptor 2

## Appendix A

**Table A1:** Detailed statistical results for the study outcomes.

Item	Statistical Test	Statistic (df)	*p* Value	Effect Size (η^2^/ε^2^)
Liver weight	One-way ANOVA	F(3, 36) = 48.166	<0.0001	0.801
Liver to body weight ratio	One-way ANOVA	F(3, 36) = 41.960	<0.0001	0.778
AST	One-way ANOVA	F(3, 36) = 6.489	0.0013	0.351
ALT	One-way ANOVA	F(3, 36) = 5.786	0.0025	0.325
Albumin	One-way ANOVA	F(3, 36) = 10.094	<0.0001	0.457
Total bilirubin	One-way ANOVA	F(3, 36) = 19.458	<0.0001	0.619
Histological score	Kruskal-Wallis	H(3) = 28.173	<0.0001	0.699
PCNA	One-way ANOVA	F(3, 36) = 27.737	<0.0001	0.698
Cyclin D1	One-way ANOVA	F(3, 36) = 19.142	<0.0001	0.615
A_2A_R	One-way ANOVA	F(3, 76) = 11.774	<0.0001	0.317
cAMP	One-way ANOVA	F(3, 76) = 8.157	0.00009	0.244
HGF	One-way ANOVA	F(3, 76) = 12.853	<0.0001	0.337
p-c-Met/c-Met	One-way ANOVA	F(3, 36) = 15.466	<0.0001	0.563
VEGF	One-way ANOVA	F(3, 76) = 15.902	<0.0001	0.386
p-VEGFR2/VEGFR2	One-way ANOVA	F(3, 36) = 16.265	<0.0001	0.575

Continuous variables were analyzed using one-way ANOVA followed by Duncan’s multiple range test. Histological scores were analyzed using the Kruskal-Wallis test followed by Holm-adjusted pairwise comparisons. Effect sizes are reported as η^2^ for one-way ANOVA and ε^2^ for the Kruskal-Wallis test.

**Table A2:** Resected liver weight at the time of partial hepatectomy (mean ± SEM).

Parameter	PHx Surgical Cohort (n = 40)
Resected liver weight (g)	6.71 ± 0.07
